# Fatherhood and breastfeeding: a qualitative exploration of counselling experiences

**DOI:** 10.1186/s13006-025-00730-8

**Published:** 2025-05-13

**Authors:** Ingvild Lande Hamnøy, Marianne Kjelsvik, Anne Bergljot Baerug, Berit Misund Dahl

**Affiliations:** 1 Norwegian University of Science and Technology, Ålesund, Norway; 2 The Norwegian Directorate of Health, Oslo, Norway

**Keywords:** Fathers, Breastfeeding family, Breastfeeding counselling, Midwives, Public health nurses, Healthcare professionals, Qualitative study, Individual interviews, Qualitative content analysis, Breastfeeding support

## Abstract

**Background:**

Including fathers as breastfeeding facilitators and providing qualified breastfeeding counselling from healthcare professionals are key factors that increase breastfeeding outcomes. It is essential to explore how healthcare professionals can effectively support fathers in navigating their roles and addressing the challenges they encounter to promote breastfeeding. We aimed to explore fathers’ experiences of being in a breastfeeding family with a particular focus on their interactions with midwives and public health nurses during breastfeeding counselling. The purpose was to gain deeper insights into the phenomenon of breastfeeding counselling from the father’s perspective to develop knowledge that can better help fathers support breastfeeding mothers.

**Methods:**

A qualitative content analysis with a phenomenological hermeneutic approach was employed using individual interviews with ten fathers in Norway between January and October 2022. The study adhered to the COREQ Checklist for reporting.

**Results:**

The meaning of fathers` experiences of being in a breastfeeding family and receiving breastfeeding counselling was formulated into three interrelated themes: being a caregiver and breastfeeding facilitator, meaning to be prepared for the father’s role and supporting mother and child; being part of a breastfeeding family which means being included or excluded in breastfeeding counselling, desire to nurture parent-child bonds and develop resilience as a couple. Managing everyday life means making their own decisions, and competent breastfeeding counsellors provide security and trust, while inadequate counselling leaves fathers feeling trapped in a chaotic situation.

**Conclusions:**

To enable fathers to fulfil their roles as caregivers and breastfeeding facilitators, healthcare professionals should actively encourage their participation and involvement in breastfeeding counselling and discussions regarding breastfeeding. Additionally, to help fathers navigate daily life confidently, healthcare professionals must offer qualified breastfeeding counselling, be aware of their needs and provide appropriate support. Empowering fathers to support breastfeeding may contribute to promoting breastfeeding, closer relationships and better health for the family.

## Background

The inclusion of fathers as breastfeeding facilitators and qualified breastfeeding counselling from healthcare professionals are key factors that increase breastfeeding outcomes [1–3]. The benefits of breastfeeding are well-documented, contributing to societal improvements in health, the economy, and the environment in low-, middle-, and high-income countries worldwide [3, 4].

Several factors influence breastfeeding practices, including legal and policy determinants, as well as the support provided by healthcare providers and family members [3, 5]. To ensure healthcare professionals are competent in providing qualified breastfeeding counselling, the `Baby-Friendly Hospital Initiative (BFHI) and the Ten Steps to Successful Breastfeeding were launched by the World Health Organization (WHO) and United Nations Children’s Fund (UNICEF) in 1991, and revised in 2017 [6]. Notably, steps three and ten recommend including families in discussions regarding the importance of breastfeeding during pregnancy and providing partners with post-discharge support [6].

Nevertheless, fathers can find the transition to parenthood stressful, with breastfeeding potentially adding to their stress as they may feel uncertain about their responsibilities towards their child [7]. Breastfeeding may also challenge fathers’ bonding with their children, leading to feelings of exclusion from the mother-child relationship [8]. A study on a breastfeeding program found that partners of breastfeeding mothers strive to be included in the family and manage daily responsibilities. Hence, breastfeeding counselling, guided by the BFHI and involving dialogue with healthcare professionals about breastfeeding, can enhance their sense of support and inclusion in the family [9].

Fathers perceive themselves to be members of the breastfeeding family [10], for this paper, a breastfeeding family is defined as a father, a child, and a breastfeeding mother. Breastfeeding counselling should adopt a teamwork approach to enable fathers to learn how to support and respond to mothers’ needs sensitively, fostering mutual respect and parental autonomy [11]. A teamwork approach contributes to longer breastfeeding durations, improves the relationship between parents, and positively impacts a child’s development [11]. In the healthcare setting, the Family-centred care (FCC) model emphasises collaborative partnerships among patients, families, and healthcare professionals and aims to promote health and well-being by empowering families to control and participate in care for their family [12, 13], in this context fathers caring for the breastfeeding mother and their child.

Both first-time and experienced fathers benefit from professional support during pregnancy and the child’s first year, underscoring the importance of their inclusion by healthcare professionals [14]. Support from healthcare professionals bolsters fathers’ confidence, enabling them to effectively assist their partners during the postnatal period. Fathers appreciate healthcare professionals who address both parents, inquire about their well-being, and encourage questions [15].

Fathers play multifaceted roles in the breastfeeding process beyond being mere facilitators. They actively participate in decisions regarding their child’s nutrition and bear responsibility for their families [16]. Despite fathers considering themselves equal parents to their newborns, some report feeling relegated to a supporting role by healthcare professionals during hospital stays, leading to insecurity regarding their roles. Early discharge home without adequate preparation exacerbates anxiety in fathers concerning the mother and child, especially when they are grappling with breastfeeding [17]. Fathers require factual and specific guidance on supporting breastfeeding mothers, including learning about the benefits of breastfeeding and understanding their role in providing encouragement and ensuring adequate rest [18].

Fathers can also experience exclusion by public health nurses (PHNs) in child health clinics, often feeling marginalised outside the mother-baby-PHN triad [19]. They have expressed a need for information on breastfeeding and infant care, as they are typically less familiar with child health services and require detailed knowledge to confidently engage with healthcare professionals [19]. It is essential to explore how healthcare professionals can effectively support fathers in navigating their roles and addressing the challenges they encounter to promote breastfeeding.

The study aimed to explore the experience of fathers within breastfeeding families, with a particular focus on their interactions with midwives and PHNs during breastfeeding counselling. The purpose was to gain deeper insights into the phenomenon of breastfeeding counselling from the father’s perspective to develop knowledge that can better help fathers support breastfeeding mothers.

## Methods

### Design

This study employed a qualitative design with a hermeneutic and phenomenological approach to investigate the meaning of fathers’ experiences with breastfeeding and breastfeeding counselling. Data were analysed using qualitative content analysis following Graneheim and Lundman [20]. Qualitative content analysis aligns with phenomenological and hermeneutic approaches as manifest content represents the concrete phenomenological description of fathers’ lived experiences, formulated as codes and categories, and the latent content aligns with the hermeneutic interpretation, expressed as themes representing the underlying meaning of the text [20, 21].

### Study setting

In Norway, children are usually born in public hospitals where parents receive breastfeeding counselling [22]. Furthermore, midwives and PHNs working at community child health clinics are responsible for offering breastfeeding counselling to families after discharge from the hospital during the postnatal period. A standardised program includes one home visit from the midwife and one from a PHN, followed by consultations at a child health clinic where both parents are invited [23]. Additionally, ‘Ammehjelpen’ is a mother-to-mother organisation in Norway that provides information and breastfeeding counselling alongside the public health service [24].

To protect and promote breastfeeding in Norway, the BFHI has been adapted and integrated into the child health service, advocating that both parents receive information about breastfeeding [25]. Research has demonstrated that these ‘Baby-Friendly’ community health clinics can increase breastfeeding rates for exclusive breastfeeding for up to 6 months [26].

Longer parental leave extends the duration of breastfeeding [27]. In Norway, both parents are entitled to paid parental leave for 12 months. Of these, 16 weeks can be allocated by the family themselves, whereas 15 weeks are reserved for the father, allowing them early involvement in the care of their child [28].

### Recruitment and participants

Participants were selected through a purposive sampling strategy combined with snowball sampling of fathers with experiences of having a breastfed child [29]. The inclusion criteria included fathers with a child born within the last 2 years, after the 37th week of pregnancy, who were breastfed exclusively or partly when they left the hospital after birth. Fathers were required to speak Norwegian and live with their children’s mothers. Leaders of community health clinics in small and large municipalities were contacted to disseminate information to the PHNs in the different child health clinics. The PHNs then approached eligible fathers, assessed their interest in participating in the study, and obtained permission to share their contact details with the first author. Three fathers initially agreed to be contacted by the first author and wished to participate in the study. Two other fathers were recruited through professional networks and meetings, where the first author informed PHNs about the study and subsequently invited the interested fathers to join the study. The remaining five individuals were recruited using snowball sampling. Overall, ten fathers from five counties and nine different child health clinics volunteered to participate in the study. Their ages varied between 30 and 50 years. Six of them had one child, whereas four had two or more children. The age of the youngest child in the family ranged from 3 to 22 months. No fathers withdrew from the study.

### Data collection

An interview guide with open-ended questions was developed to understand fathers’ experiences of receiving breastfeeding counselling from midwives and PHNs as members of the breastfeeding family. The interview guide was evaluated by two fathers with experience in having a breastfeeding child prior to data collection. Minor adjustments were made to clarify the questions and ensure that the target questions were answered. Examples of questions in the interview guide were as follows: ‘How did you experience the breastfeeding situation when the mother and child came home from the hospital?‘, ‘Can you tell us about a challenging situation related to breastfeeding that you experienced?‘, ‘As a father, how are your experiences with receiving counselling from midwives and PHNs about breastfeeding?‘, and ‘What do you feel are the most important experiences you have gained as a father related to breastfeeding and counselling on breastfeeding?‘.

The first author conducted ten individual interviews with fathers in Norway between January and October 2022. The participants could choose between meeting for the interview in person or being interviewed digitally. Eight interviews were conducted virtually (Microsoft Teams), whereas two were in a private meeting room in a public office. The interviews lasted 40–70 min, with an average of 57 min, and were audio-recorded and transcribed verbatim by the first author. Data collection continued until the authors found that the data were rich and sufficiently dense to describe the phenomena [29].

### Data analysis

The data were analysed using Graneheim and Lundman’s method of qualitative content analysis [20]. Each transcribed interview was read several times by the authors to obtain a comprehensive understanding. Subsequently, each interview was divided into meaning units, which were condensed, abstracted, and labelled with specific codes by the first author. These codes were compared and sorted into categories based on similarities and differences, representing the manifest content. To increase the understanding of the data, codes and categories were discussed by all the authors. Furthermore, the categories were revised and compared across all interviews, considering the entire text, meaning a back-and-forth movement between the whole and the parts [20]. A reflective process and discussions between the authors were conducted to determine the underlying meaning and latent content of the texts, resulting in the formulation of three interrelated themes representing the meaning of the phenomena of breastfeeding and breastfeeding counselling (Examples from the analysis in Table 1).

**Table 1:** Examples from the analysis

Meaning Unit	Condensed Meaning Unit	Code	Category	Theme
*´You just have to show up*,* it’s not a job once*,* you just have to do it*,* you have no other choice*,* and when you wake up time and time again feeling dizzy. You just must be strong and then it will pass*,* […]. You must show up because there is a child who is completely dependent on you.´(F8)*	You must show up and be strong when a child is dependent on you	Be helpful and contribute	A sense of responsibility in supporting the mother and child	Being a caregiver and breastfeeding facilitator
*´He could not take milk from his mother from the start*,* he was quite small. Then I got involved*,* it was a steep learning curve with cup feeding then [.]. It was a nice experience for me to contribute with what I could do. […]. I got involved in most of it*,* both with weighing and cup feeding.*´(F3)	It was fulfilling to contribute; I participated in weighing and cup feeding	Involved	To be included or excluded	Being part of a breastfeeding family
*´The help we got was invaluable*,* I dare say that I think it helped a lot for the mother and me. It helped us to get through it and persevere until it would work out. There were signs that it was possible to master breastfeeding when both I and the PHN helped[.]. Mother had given up long before without the response from the PHN.´(F9)*	The assistance we received was invaluable in helping us persevere	Effective breastfeeding support	Competent breastfeeding counsellors provide security and trust	Managing everyday life

### Ethical considerations

The study received ethical approval from Sikt on 29 June 2021 (No. 784292). Following the Declaration of Helsinki [30], all fathers received written oral information about the study before consenting to participate. They were informed of their right to withdraw from the study without any consequences before the analysis and publication. Interview data were de-identified during the transcription and stored securely to ensure confidentiality.

### Rigour and reflexivity

Rigour and trustworthiness in this study were maintained by applying the criteria of credibility, dependability, confirmability, and transferability [31]. The fathers were informed of the first author’s background as a PHN, which contributed to gaining trust and a better understanding of their descriptions. Reflections on how the first author’s pre-understanding as a PHN could influence the understanding and interpretation of the findings were important to ensure the credibility of the study. To ensure credibility, the analysis was clearly described, and the codes, categories, and themes were discussed between the authors, including three RNs, two PHNs, and one nutritionist. The first author is a Ph.D. student and the others have a Ph.D. Direct quotes from fathers were cited to help readers distinguish between the fathers’ voices and the author’s interpretation of their experiences. Dependability was achieved using the same interview guide for all fathers, with only minor adjustments during the data collection period. To ensure a reflective attitude and confirmability of the study, the first author’s reflections were written immediately after the interviews, during the transcription, and in the overall reading of each interview and were subsequently discussed by the authors. The setting, participants, and research process were described to facilitate transferability. The use of the Consolidated Criteria for Reporting Qualitative Research (COREQ) provided transparency for this study [32].

## Results

The meaning of fathers’ experiences of being in a breastfeeding family and receiving breastfeeding counselling was formulated into three interrelated themes: being a caregiver and breastfeeding facilitator, being part of a breastfeeding family, and managing everyday life as illustrated in Table 2.

**Table 2:** Description of themes and categories

Themes	Categories
Being a caregiver and breastfeeding facilitator	The need to be prepared for the father’s role
	A sense of responsibility in supporting the mother and child
Being part of a breastfeeding family	To be included or excluded
	Desire for a close relationship with the child
	Develop resilience as a couple
Managing everyday life	The importance of making your own decisions
	Competent breastfeeding counsellors provide security and trust
	Feeling trapped in a chaotic situation due to inadequate breastfeeding counselling

### Being a caregiver and breastfeeding facilitator

This theme was formulated from two categories: the need to be prepared for the father’s role and a sense of responsibility in supporting the mother and child.

### The need to be prepared for the father’s role

Fathers expressed their desire to care for mothers and children during breastfeeding. However, this requires them to be prepared on how breastfeeding works and familiar with the challenges that could arise during the breastfeeding period.

Some fathers in this study reported preparing for fatherhood and facilitated breastfeeding by reading books or web pages. Some attended prenatal courses online but learned little about breastfeeding and mostly about childbirth. A father emphasised the importance for fathers to understand that difficulties with breastfeeding are normal. Thus, they could better support mothers who felt pressured to breastfeed or inadequate due to breastfeeding problems, including the need to reduce or stop breastfeeding:

*It’s important to be aware of it so that you can clearly tell the mother that it is normal*,* most people struggle with breastfeeding and if it doesn’t resolve and doesn’t go away*,* that’s also completely okay. […]. You must be a filter between the internet and the mother.* (F2)

Having realistic expectations about post-birth challenges, such as sore nipples, discomfort, and sleep deprivation, could reduce shock for both parents in the first week and increase fathers’ understanding of the mother’s situation. As preparation, fathers can gain an understanding of their role during the breastfeeding period. Some fathers expressed the desire to help and support mothers without knowing how to effectively contribute, which was described as difficult. A father said that he felt helpless seeing the mother tired from breastfeeding and wanted to support her, but was uncertain how he could help. He asked for more information from midwives and PHNs about what was expected of fathers during the breastfeeding period and counselling about how they could contribute to the child and mother.

### A sense of responsibility in supporting the mother and child

The fathers felt responsible for their families and described having an essential role as facilitators, enabling mothers to focus on breastfeeding. Examples included arranging practical tasks at home, such as giving the mother breakfast or taking care of siblings so she could sleep, relax, and eat or drink between breastfeeding sessions. A participant highlighted the importance of active involvement in supporting mothers and the child:

*I think it has given me a lot to help with what I can*,* always involve myself as much in my children as I can*,* otherwise*,* it will be very…*,* just sit and watch mother arrange[.]*,* the most important experience is taking responsibility*,* doing it*,* change diapers*,* picking up things.* (F3)

Fathers maintained that actively contributing to the family during the breastfeeding period had become part of the contemporary male role in society, though parents had different roles. Although they felt responsible for supporting the mother, their role remained in the background, helping mothers and children when needed.

Fathers emphasised the importance of being involved and learning about breastfeeding, as well as recognising children’s normal cues and sleeping patterns, to share the responsibility of ensuring that the baby receives enough food. An experienced father underlined the importance of recognising signals from the baby in case something happened to the mother. To support and care of the mother, fathers needed to spend time with their families. Knowledge of breastfeeding would help them appreciate that it is both an emotional and technical exercise for mothers and children.

Most fathers expressed that taking responsibility was positive, although some found it demanding and exhausting. Some worried about their partners, who were vulnerable and tired after delivery, slept little, and struggled to learn to breastfeed. A father felt that his family was dependent on him, and he had to be strong and find inner strength that he did not know he had.

*You just have to show up*,* it’s not a job once*,* you just have to do it*,* you have no other choice*,* and when you wake up time and time again feeling dizzy. You just must be strong and then it will pass*,* […]. You must show up because there is a small child who is completely dependent on you.* (F8)

Another father described how he could not sleep because he felt responsible for watching over their child when the mother slept during breastfeeding, worrying the child could be left in a dangerous situation.

Some fathers felt torn between family responsibilities and their jobs, stretching far to provide support despite the challenges. A father explained how he struggled to support a tired mother with breastfeeding while managing his work duties:

*I was constantly exhausted because we had agreed that when he did not need breastfeeding*,* I could also help*,* but when I had to give him a bottle in the middle of the night and make him sleep again*,* and then take care of him early in the morning before going to work*,* that was simply too much. (F9)*

Good support and collaboration with their PHN helped him navigate these responsibilities. The PHN acknowledged the father’s experiences and competency in breastfeeding, offering additional follow-up when needed.

### Being part of a breastfeeding family

The second theme encompassed three categories: to be included or excluded, desire for a close relationship with the child, and develop resilience as a couple.

### To be included or excluded

The fathers described the need to be seen as part of the family and as collaborative partners in addressing breastfeeding problems. Some fathers reported that hospital nurses facilitated their collaboration with the mother and child immediately after delivery. When allowed to be present in the maternity ward, fathers felt acknowledged as equal caregivers, were able to connect with their children, and found fulfilment in supporting the mother. A father had a positive experience of being included when he was taught how to feed the baby milk from a cup before breastfeeding was established and took responsibility for helping the child gain weight after discharge from the hospital:

*He could not take milk from his mother from the start*,* he was quite small. Then I got involved*,* it was a steep learning curve with cup feeding then [.]. It was a nice experience for me to contribute with what I could do. […]. I got involved in most of it both with cup feeding and weighing.* (F3)

Another father described how midwives and PHNs made him feel recognised by speaking with him, making eye contact, treating him seriously, and taking time to give him information adapted to what he needed. Fathers needed both mothers and midwives/PHNs to acknowledge their fatigue and the challenges when breastfeeding difficulties occurred, emphasising the need to support and assist the mothers.

The coronavirus disease pandemic significantly influenced the likelihood of some fathers meeting their newborns. Although some were allowed to be with their families in the hospital, others were restricted from visiting because of the pandemic protocols to avoid the spread of the virus. Fathers desired to be with their families in the hospital from the beginning, believing it was essential for breastfeeding. They perceived an increased risk of breastfeeding problems if mothers were alone with their child in the first vulnerable days post-delivery, finding exclusion difficult. Fathers could be miles away waiting for their family to come home and relied solely on phone calls to connect with the mother; thus, fathers described feeling helpless about not being there for their family. A father described how he felt ‘kicked out’ from the hospital, lamenting the lost bonding time with his child after delivery. Fathers explained that they missed opportunities to learn about breastfeeding and nutrition for the child both in the hospital and after discharge from the hospital. Due to pandemic rules, some home visits were replaced by consultations at child health clinics, where fathers were not permitted to be present. One father perceived that he had not received any breastfeeding counselling when he had his second child:

*As a father during the corona-time*,* I have to think*,* no. I was not allowed to join. No*,* there was no breastfeeding counselling. (F1)*

On the other hand, fathers who were allowed to attend consultations felt excluded if they were not included in the discussions about breastfeeding. A father reported that breastfeeding received little attention during home visits. Another father did not feel included in conversations about breastfeeding by the PHN who visited them:

*I had some questions*,* (.)*,* but I remember sitting with the feeling that my question didn’t have any relevance or meaning for breastfeeding*,* even though I stood by it and wanted to try to help*,* move our child into the right position to latch on the nipple. I think some of the questions were relevant*,* but I remember feeling based on the feedback I got*,* it was not so important*,* because the important thing is what mother means. After all*,* it is she who must breastfeed (.). I concluded; this visit is the most for the mother*,* it’s not for me.(F5).*

### Desire for a close relationship with the child

Fathers highlighted that they wished to spend time getting to know their children. A father described the wonderful feeling of contact he experienced during the first days with his infant:

*I will never forget those days. Unbuttoning my shirt*,* lying in bed—she was lying on my chest*,* wearing a nappy and covered with a blanket. She just lay there for hours*,* enjoying herself. I felt I got to spend more time with her*,* it was just the two of us at the start.* (F5).

However, they had varied experiences regarding how breastfeeding affected the possibility of spending time with their child and developing relationships with them. Most fathers felt prepared to take a supportive role in the first month and found it natural, whereas others found it difficult. For some fathers, breastfeeding reduced their opportunity to bond with their child as the mother handled all feeding, leading them to feel excluded from the mother-child relationship. They sought ways to cope by taking on useful tasks such as housework or cooking, enabling the mother to relax when she was not breastfeeding. Lack of inclusion by midwives and PHNs in breastfeeding counselling during the establishment of breastfeeding or when breastfeeding difficulties occurred reinforced the feeling of being outside the mother-child relationship.

However, fathers appreciated opportunities to care for the child when the mother was pumping milk or resting. They valued midwives and PHNs who encouraged their participation and questions, which empowered them to be actively involved with their children and their families. For some fathers, breastfeeding reduced daily stress and provided more bonding time with their children compared to bottle-feeding, which meant much work with preparing milk and washing bottles. A father who had experienced both bottle-feeding and breastfeeding with his children described how breastfeeding facilitated a close relationship with his child:

*I feel it was just stress with the bottle*,* yes*,* you get involved*,* but you hold a child and put something in their mouth*,* [.] I don’t know if you can call it involved[.]. I felt that it was just working because you know when you’re done you have to wash*,* cook*,* pump*,* and prepare formula*,* so achieving 100 per cent full breastfeeding is so worth it. [.]. (F10)*

### Develop resilience as a couple

Fathers emphasised the importance of couple resilience in navigating breastfeeding situations. All our study participants used the term ‘*we’* when referring to breastfeeding, with one father describing ‘*breastfeeding as a two-person job’ (F4)*. Some families managed more independently than normal if they experienced inadequate follow-up and assistance from the healthcare system, whereas others *chose* alternative resources instead of counselling from midwives or PHNs to solve the challenges they faced. Parents used digital media/webpages to find information and establish parent groups and visited open kindergartens for observational learning from other parents/children, whereas others sought support from grandparents and friends. Some families opted not to seek additional counselling from midwives and PHNs beyond the standardised program, preferring to manage breastfeeding together. They believed in their ability to problem-solve even as beginners and relied on each other to find solutions.

*When we first managed to start breastfeeding*,* it was a bit tiring to deal with*,* and we just wanted to figure things out ourselves and of course*,* contact the child health clinic when we wanted to*,* but we did not really want to go there in a way.*(F7).

However, fathers expressed the importance of seeking counselling and support at a child health clinic, if needed.

### Managing everyday life

Three categories were associated with this theme: the importance of making your own decisions, competent breastfeeding counsellors provide security and trust and feeling trapped in a chaotic situation due to inadequate breastfeeding counselling.

### The importance of making your own decisions

Most fathers stated that they preferred the advice of midwives and PHNs on their child’s nutrition. Nevertheless, some families wanted to make their own feeding choices and trusted sources other than midwives and PHNs. Consequently, they did not always consult midwives or PHNs about their decisions. A father described how their PHN recommended reducing breastfeeding at night once solid foods were introduced, but they disagreed and continued to breastfeed without informing the PHN. These parents had much experience with breastfeeding, believing prolonged breastfeeding at night benefitted their child. They also cited research they had read, which they considered more relevant and up-to-date than the information provided by the PHN:

*You trust the PHN*,* you do*,* but when you know that the recent research says different things you try to say it. But I think it is very difficult to argue against a professional if you have read something else in an article yourself*,* it is very difficult.* (F10)

Being honest with midwives and PHNs about their experiences with breastfeeding counselling could be difficult for fathers. Although some fathers reported that they did not receive qualified breastfeeding counselling from all midwives and PHNs, they hesitated to express their dissatisfaction with the help offered. Our study participants were careful in describing the midwives and PHNs they encountered in negative ways, often portraying them as kind and helpful despite noting deficiencies in counselling and continuity in the follow-up. Relating to ten different PHNs, a father stated refraining from criticising them directly but admitted feeling exhausted by the experience. Another father whose child struggled with weight gain described feeling vulnerable and uncomfortable during clinic visits to weigh their child in an open area crowded with people yet downplayed these feelings, acknowledging it was minor and understood the child health clinic’s procedures.

### Competent breastfeeding counsellors provide security and trust

The fathers emphasised that meeting competent, helpful, kind, and caring midwives and PHNs at the child health clinic was reassuring. They appreciated the calm atmosphere and felt welcomed as individuals. The clinic was perceived as a place where they felt normalised, as staff wore ordinary clothing. Fathers felt safe when the midwives and PHNs assured them that their babies were healthy, confirmed their parenting decisions, and showed that they had confidence in managing everyday life with their children.

When fathers experienced good breastfeeding counselling and support, it helped the family navigate their breastfeeding situation. One family encountered initial breastfeeding problems, and the father emphasised that the support they received from a patient PHN was essential in helping them persist until the mother and child mastered breastfeeding:

*The help we got was invaluable*,* I dare say that I think it helped a lot for the mother and me. It helped us to get through it and persevere until it would work out. There were signs that it was possible to master breastfeeding when both I and the PHN helped [.]. Mother had given up long before without the response from the PHN.* (F9)

Meeting midwives and PHNs who demonstrated knowledge of breastfeeding and the ability to convey good advice made fathers feel safe:

*The most important thing was that she is a very nice woman who understands people. She is good at explaining*,* appears trustworthy*,* and makes people relax. She sat back and waited because the stress level with breastfeeding was very high. So just puh… She explained things*,* and things fall into place. (F7)*

A father distinguished between ordinary midwives and PHNs and what he described as ‘breastfeeding experts’, noting their competency in breastfeeding. The midwives/PHNs were perceived as experts when they offered clear answers to their questions, practical advice on breastfeeding, were solution-orientated, updated, engaged, had a lot of experience, and specialised in breastfeeding: *‘It was very nice to have someone with experience who could help us through it*.’ *(F6*). In contrast, midwives and PHNs only offered general advice and said that everything was normal.

The fathers appreciated that their advice aligned with their readings from other sources. Good information on breastfeeding and tips on quality-assured webpages were helpful. Over time, support from midwives and PHNs to reduce pumping and bottle feeding while increasing breastfeeding frequency gave parents more control over the situation, confidence to continue trying to breastfeed, and contributed to their breastfeeding success.

### Feeling trapped in a chaotic situation due to inadequate breastfeeding counselling

In contrast to experiencing qualified breastfeeding counselling, some fathers perceived that inadequate support provided to their mothers or families led to breastfeeding problems. This made managing daily life and the transition to fatherhood more challenging than expected. Fathers found it frustrating and confusing to receive conflicting professional advice on breastfeeding problems, making it difficult to decide how to handle these situations:

*There was a bit of conflicting advice then*,* it was the general practitioner who said you must squeeze and massage*,* then the staff in the maternity ward said that’s the worst thing you can do*,* then it gets more inflamed. It was extremely frustrating. Different professionals*,* who ideally should have provided consistent advice*,* were not at a slight difference—it was like they were at opposite ends of the scale when it came to solutions.* (F4)

Some fathers perceived breastfeeding knowledge seemed random and not systematic within healthcare services. They felt their families were left to find solutions to their problems. For instance, inadequate counselling could lead to a demanding situation where mothers were advised to frequently pump to increase milk production without clear guidelines, potentially leading to overproduction of milk, breast swelling, and mastitis. Another difficult situation was when parents were encouraged by hospital nurses that the child should be breastfed every third hour instead of adjusting breastfeeding after the baby’s cues. Thus, parents tried to handle a demanding feeding regime at home with bottle feeding in addition to breastfeeding. Since some of these families had to switch between pumping, breastfeeding, and bottle feeding, some fathers felt like assistants, spending significant time formulating and washing bottles and equipment. These demanding first weeks left them questioning what fatherhood would entail. A father asked himself if he would ever enjoy mountain hikes again. Another father said that it was unpleasant when everyday life was characterised by misery because of breastfeeding difficulties. It was a great relief for him when the mother decided to stop breastfeeding allowing their family to regain a sense of normalcy:

*I was just really relieved and happy that it was finally over*,* breastfeeding was just trouble*,* just problems and negativity then.* (F4)

Breastfeeding problems also strained the parents’ relationship. A father described that although minimal interaction was expected during the infancy period, breastfeeding problems made survival and daily life more dominant than fostering a relationship.

## Discussion

In our study, fathers wished to participate in a breastfeeding family and take up responsibilities for their mothers and children. Midwives and PHNs were expected to include them in breastfeeding counselling to contribute to breastfeeding, support, and care for the mother, and develop a close relationship with their child. However, some fathers felt excluded by midwives and PHNs and experienced inconsistencies in the quality of counselling provided to the family. These experiences affected how they mastered their fathers’ roles and everyday life.

In our study, fathers emphasised that supporting the breastfeeding process integral to their role as fathers. They underlined the importance of being included in breastfeeding counselling to actively support breastfeeding and reduce the risk of breastfeeding problems. This aligns with previous research by Koksal et al. [1], Panahi et al. [2], and Rollins et al. [3], who demonstrated that including fathers in breastfeeding counselling increased breastfeeding rates. Fathers also expressed the desire to spend time with the mother and child, providing support and care for the mother. However, several fathers in the study were excluded from the hospital and partly from the child health clinic, missing opportunities to meet midwives and PHNs for breastfeeding counselling. It became challenging to support the mother with breastfeeding, and reduced the time with their families. During the pandemic, some fathers were excluded from the consultations in our study because of the restrictions. Still, the findings highlight the importance of allowing the presence of fathers in hospitals and inviting them to consultations at the child health clinic, aligning with BFHI in hospitals and guidelines for ‘Baby-friendly community health clinics’ [6, 25].

Nevertheless, fathers in the study who were allowed to join consultations could still feel excluded by the midwives and PHNs, either because breastfeeding was not discussed or because their questions were perceived as irrelevant. Similarly, the research by Feenstra et al. [17] and Høgmo et al. [19] revealed that fathers can feel excluded by healthcare professionals in dialogues about breastfeeding, highlighting that mere presence in consultation does not ensure inclusion. Palmèr and Gustafsson [33] suggested that women should be encouraged to tell their breastfeeding stories and describe the meaning of breastfeeding as a starting point for breastfeeding care and a foundation for a trusting relationship with healthcare professionals. Considering the participants’ desire to be acknowledged by midwives and PHNs, fathers should also be encouraged to describe their experiences and wishes for breastfeeding to ensure their inclusion in dialogues about breastfeeding and understand their experiences in the breastfeeding family.

Fathers in our study described feeling included when midwives and PHNs made eye contact with them, gave them practical tasks, such as cup feeding, and took their questions seriously. To facilitate participation in practical care helps fathers feel acknowledged and respected as caregivers, which is important for effective collaboration between families and healthcare professionals in the FCC model [13]. They stated that they needed more information about breastfeeding adapted to their needs and circumstances, normal baby behaviours, and their role when the mother was breastfeeding. This aligns with the findings of Sihota et al. [8], Earle and Hadley [18], and Panahi et al. [2], who emphasised that fathers require practical and direct information about breastfeeding and guidance on understanding the mother’s needs and baby’s cues. Information sharing is one of the prerequisites for families to participate in care [13], thus, midwives and PHNs can facilitate fathers’ participation in breastfeeding families by sharing customised information with them.

Our study participants felt responsible for facilitating breastfeeding, and supporting and caring for breastfeeding mothers and children. Nevertheless, some found daily life chaotic and demanding responsibility. A lack of breastfeeding counselling or conflicting advice exacerbated their sense of chaos and loss of control, making the transition to fatherhood more challenging. Fathers excluded from breastfeeding discussions felt helpless and worried, hindering their ability to support mothers breastfeed effectively. Similarly, Shorey et al. [7] and Feenstra et al. [17] demonstrated that fathers can struggle with stress and anxiety when unsure how to fulfil their responsibilities in supporting the breastfeeding mother. Delmar et al. [34] investigated how patients experience the meanings of autonomy when meeting healthcare professionals, noting that expectations placed on patients to be responsible and autonomous can be overwhelming. Even though fathers are not patients, fathers of newborn children can be in stressful situations with expectations of taking responsibility for their child, according to Shorey et al. [7]. Therefore, fathers who struggle with their responsibilities and challenges of fatherhood may feel insecure and overwhelmed. Examples of this were fathers who described being exhausted, worried, and unable to sleep well because the family depended on them. Fathers in the study expressed a need for counselling from midwives and PHNs on how to support their mothers in challenging situations related to breastfeeding. Qualified breastfeeding counselling from knowledgeable midwives or PHNs helped families feel safe, find solutions, and navigate the situation effectively, in contrast to poor breastfeeding counselling. An example of this was a father who described that meeting a midwife with experience in breastfeeding was helpful and made him feel safe in the breastfeeding support role and in control of the feeding situation. Thus, to support fathers in finding a balance between autonomy and the need for support and counselling, midwives and PHNs should be available to help the family as needed. They should engage in their situation and daily life, acknowledge their needs and feelings, and be aware of how much support each father needs to manage daily life. This approach may help fathers feel more confident about their roles within the breastfeeding family and encourage active participation in caring for their families. Consequently, it can promote the health of fathers and their families, aligning with the goals of the FCC model [13]. However, Kokorelias et al. [12] highlighted the need for developing procedures for implementing FCC in practice in different health settings and suggested further research on how the FCC model can improve the caring for individuals and families, particularly in the context of breastfeeding counselling.

### Strengths and limitations of the work

This study faced limitations related to recruitment and variability. Including younger fathers could have broadened the range of experiences with fatherhood, breastfeeding, and counselling. Nevertheless, the study provides diverse insight into receiving breastfeeding counselling from midwives and PHNs, encompassing perspectives from both first-time and multiple fathers. Although snowball sampling may have restricted the network of fathers, it facilitated trust-building for the first author, as participants were recommended by acquaintances.

All researchers were female. Involving male researchers could have strengthened the study, and might be done in future research to give a deeper understanding of the meaning of fathers` experiences within breastfeeding families.

Our study was conducted in Norway, where parental leave legislation facilitates fathers’ involvement in childcare. This can influence both fathers’ and society’s expectations regarding participation in the breastfeeding process. Expectations for fathers’ involvement may vary across countries depending on their parental leave policies and cultural context. Nevertheless, this study demonstrates the importance of including fathers as facilitators of breastfeeding and caregivers for breastfeeding mothers and their children. The study highlights that qualified breastfeeding counselling and support for the dyad and the family are vital to positive breastfeeding experiences for families in all countries despite differences in breastfeeding protection and promotion polices.

Including fathers from different cultural backgrounds in our study could have contributed to greater variation in our findings, providing a broader perspective on the phenomena of breastfeeding and breastfeeding counselling.

## Conclusions

This study provides novel insights into fatherhood and breastfeeding, and fathers’ experiences of receiving breastfeeding counselling from midwives and PHNs within the context of breastfeeding support for families. Our findings highlight that fathers serve as caregivers for both mothers and children, as well as facilitators of breastfeeding. However, they require active invitation to participate in consultations and discussions regarding breastfeeding, and should be encouraged by healthcare professionals to tell their expectations and wishes for the breastfeeding of their child. It is crucial that fathers receive competent breastfeeding counselling from healthcare professionals to effectively fulfil their fathers’ role, assume responsibilities in supporting mothers and children, and navigate daily challenges with confidence. To enable this, healthcare professionals should facilitate effective collaboration with fathers by acknowledging their needs and feelings, being aware of how much counselling they need to find the balance between autonomy and need for counselling, and providing an appropriate level of support.

Empowering fathers to play a more active role in supporting breastfeeding, may contribute to promote breastfeeding, closer relationships and better health for the whole family.

This study investigated fathers’ experiences, however, partners other than fathers may also support breastfeeding mothers. Further research is necessary to investigate these diverse experiences, including those of same-sex parents. Further development of younger fathers’ experiences in breastfeeding families, and fathers’ perspectives from different social and cultural backgrounds may assist the understanding of the phenomenon of breastfeeding counselling.

## Data Availability

No datasets were generated or analysed during the current study.

## Abbreviations

BFHI: Baby-Friendly Hospital Initiative
COREQ: Consolidated criteria for reporting qualitative research
FCC: Family-centred care
PHN: Public Health Nurse
UNICEF: United Nations Children’s Fund
WHO: World Health Organization
